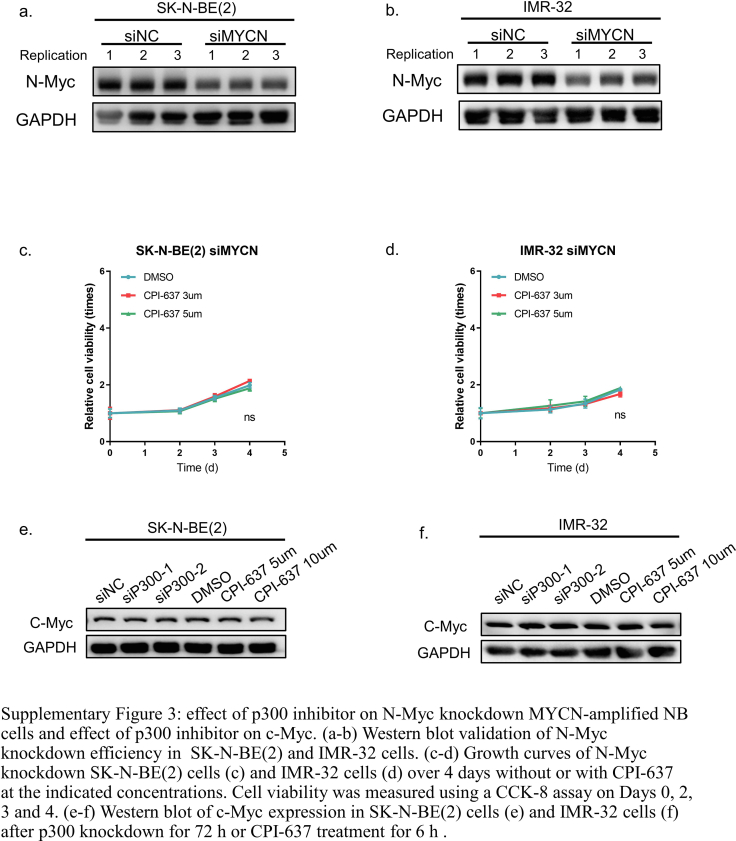# P300 Interacted With N-Myc and Regulated Its Protein Stability *via* Altering Its Post-Translational Modifications in Neuroblastoma

**DOI:** 10.1016/j.mcpro.2023.100504

**Published:** 2023-01-26

**Authors:** Cheng Cheng, Tian He, Kai Chen, Yuanxia Cai, Yaoyao Gu, Lijia Pan, Peiwen Duan, Yeming Wu, Zhixiang Wu

**Affiliations:** 1 Department of Pediatric Surgery, Xinhua Hospital Affiliated to Shanghai Jiao Tong University School of Medicine, Shanghai, China; 2 Division of Pediatric Oncology, Shanghai Institute of Pediatric Research, Shanghai, China; 3 Department of Pediatric Surgery, Children’s Hospital of Soochow University, Suzhou, China

**Keywords:** neuroblastoma, *MYCN*, post-translational modifications, interacting proteins, protein stability, BP, biological process, CC, cellular component, co-IP, coimmunoprecipitation, GO, Gene Ontology, GST, glutathione-*S*-transferase, IgG, immunoglobulin, KEGG, Kyoto Encyclopedia of Genes and Genomes, MF, molecular function, MS, mass spectrometry, NB, neuroblastoma, PTM, post-translational modification

## Abstract

*MYCN* amplification is an independent risk factor for poor prognosis in neuroblastoma (NB), but its protein product cannot be directly targeted because of protein structure. Thus, this study aimed to explore novel ways to indirectly target N-Myc by regulating its post-translational modifications (PTMs) and therefore protein stability. N-Myc coimmunoprecipitation combined with HPLC–MS/MS identified 16 PTM residues and 114 potential N-Myc-interacting proteins. Notably, both acetylation and ubiquitination were identified on lysine 199 of N-Myc. We then discovered that p300, which can interact with N-Myc, modulated the protein stability of N-Myc in *MYCN*-amplified NB cell lines and simultaneously regulated the acetylation level and ubiquitination level on lysine-199 of N-Myc protein *in vitro*. Furthermore, p300 correlated with poor prognosis in NB patients. Taken together, p300 can be considered as a potential therapeutic target to treat *MYCN*-amplified NB patients, and other identified PTMs and interacting proteins also provide potential targets for further study.

## Highlights

• P300 interacted with N-Myc and regulated its protein stability.
• P300 can simultaneously modulate the acetylation and ubiquitination levels of N-Myc.
• P300 correlated with poor prognosis of neuroblastoma patients.
• P300 regulated cell proliferation in MYCN-amplified neuroblastoma cell lines.

## In Brief

Endogenous and exogenous N-Myc coimmunoprecipitation combined with HPLC–MS/MS identified 16 PTM residues of N-Myc and 114 potential N-Myc-interacting proteins. Among them, p300 interacted with N-Myc and modulated the protein stability of N-Myc by simultaneously regulating the acetylation and ubiquitination level on lysine-199 of N-Myc protein in vitro. Furthermore, p300 promoted cell proliferation and invasion of MYCN-amplified NB cells and correlated with poor prognosis in NB patients, making p300 a potential therapeutic target for MYCN-amplified NB patients.

## Graphical Abstract

**Figure:**
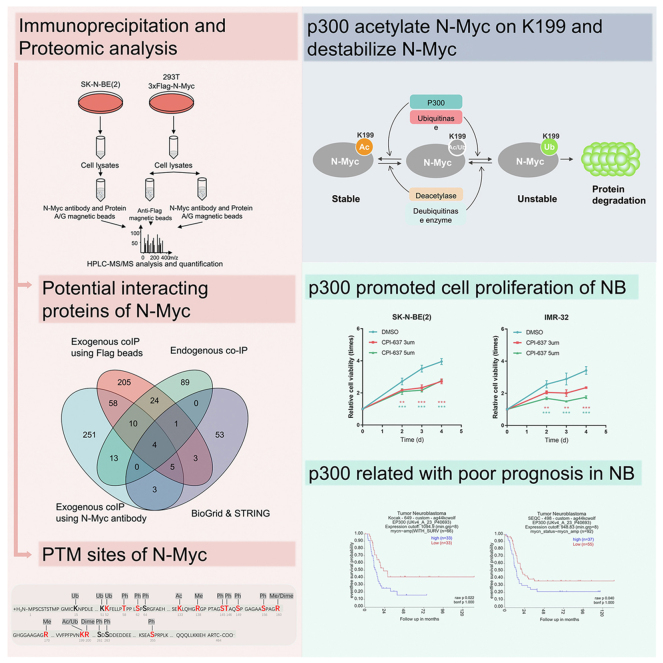

Neuroblastoma (NB) is the most common extracranial solid tumor in children, accounting for approximately 7 to 8% of childhood tumors (1) and causes 15% of deaths related to childhood malignant tumors. According to the age at the time of tumor diagnosis, histopathological type, International Neuroblastoma Staging System stage, the presence or the absence of *MYCN* oncogene amplification, and tumor DNA index, NB can be classified into low-, intermediate-, and high-risk groups, which can be used to guide subsequent treatment and evaluate prognosis (2). The prognosis of low- and intermediate-risk patients is good, with a survival rate of more than 95%, but the long-term survival rate of high-risk children is still less than 40%.

As an independent risk factor for poor prognosis, *MYCN* amplification occurs in approximately 40% to 50% of high-risk NB cases (3). Its protein product, N-Myc proto-oncogene protein (N-Myc), is a transcription factor and is considered to promote NB cell proliferation, invasion, and angiogenesis and inhibit cell differentiation, playing an important role in the occurrence and development of NB (4, 5). However, it is difficult to directly target N-Myc because its protein conformation has the characteristics of dynamic changes (6). Recently, protein post-translational modifications (PTMs) have been proved to play a vital role in regulating the protein stability and transcriptional activity of N-Myc, and corresponding regulatory enzymes can therefore provide ideal targets for indirectly modulating N-Myc (7, 8). A variety of N-Myc-interacting proteins have been confirmed to execute through regulation of its PTMs, such as AURKA (9, 10), HUWE1 (11), USP7 (12), and Trim32 (13). Thus, identifying PTMs and their regulating proteins is a promising way to reveal new strategies to regulate N-Myc and its biological function.

*EP300* encoded histone acetyltransferase p300 (p300), which can regulate transcription *via* chromatin remodeling as a histone acetyltransferase, and also functions as acetyltransferase for nonhistone targets and regulates their activity or protein stability (14). As a result, p300 influences a large number of proteins and participates in many essential cellular functions, such as proliferation, cell cycle, cell differentiation, the DNA damage response, and others (15). The overexpression or mutation of p300 has been reported to be associated with several malignant tumors (16). The small-molecule inhibitors of p300 developed rapidly in recent years, and some of them are currently under clinical trial for treatment of malignant tumors (17, 18).

In this study, we performed both endogenous and exogenous N-Myc coimmunoprecipitation (co-IP) and HPLC–MS/MS to identify PTM residues and N-Myc-interacting proteins. As a result, we identified 16 known or unknown PTM residues of N-Myc. In addition, we found that the lysine-199 residue of N-Myc has both acetylation and ubiquitination modifications, suggesting that there may be some PTM crosstalks, but it is unknown whether it will affect the function or protein stability of N-Myc. Therefore, we next used synthetic peptides to verify the existence of two modifications on the lysine-199 residue. We also identified 114 potential N-Myc-interacting proteins screening from the two of three co-IP results. Among these proteins, we found numerous Gene Ontology (GO) terms related to protein PTMs through GO enrichment analysis, which may have the function of regulating the PTM of N-Myc. We then performed experiments to screen several acetylation-related enzymes and confirmed the regulatory effect of p300 on the PTM of N-Myc *in vitro* as well as the stability of N-Myc protein *in vivo*.

## Experimental Procedures

### Cell Cultures, Transfections, and Reagents

SK-N-BE(2), IMR-32, and 293T cell lines were obtained from the American Type Culture Collection. Cell lines were authenticated by short tandem repeat profiling (GENEWIZ, Inc). SK-N-BE(2) and IMR-32 cells were maintained in a 1:1 mixture of Eagle's minimum essential medium and F12 medium (catalog nos.: 61100061/12500062; Thermo Fisher Scientific) supplemented with 10% fetal bovine serum (catalog no.: 10270106; Gibco), 100 units/ml penicillin, and 100 μg/ml streptomycin. 293T cells were cultured in Dulbecco's modified Eagle's medium (catalog no.: 10-013-CVR; Corning) supplemented with 10% fetal bovine serum, 100 units/ml penicillin, and 100 μg/ml streptomycin.

The whole length of *MYCN* with a 3xFLAG tag sequence or 3xFLAG sequence only was constructed, subcloned into a Ubi-MCS-SV40-EGFP-IRES-puromycin vector, and further constructed as a lentivirus by Shanghai GeneChem Co, Ltd. 293T cells were treated with corresponding lentivirus for 24 h. After cultured for another 48 h, 2 μg/ml puromycin was used to screen stable infected cells for 24 h, and then the infection efficiency was determined by Western blot before further use. The sequences of full-length MYCN and four deletion mutated MYCN with a 3xFLAG tag (Δ1–123, Δ382–464, Δ346–464, and Δ281–464), and FLAG sequence only were constructed and subcloned into CMV-MCS-SV40-Neomycin vector. The amino acid sequence of 3xFLAG tag we used was DYKDDDDKGDYKDDDDKIDYKDDDDK. All plasmids were constructed and provided by Shanghai GeneChem Co, Ltd. These plasmids were transfected into 293T cells using Lipofectamine 2000 transfection reagent (Invitrogen) and transiently expressed according to the manufacturer's instruction. The transfection efficiency was validated by Western blot analysis of N-Myc expression.

Remodelin hydrobromide (S7641) was purchased from Selleck, and NU9056 (HY-110127) was purchased from MedChemExpress.

### Experimental Design and Rationale

To generate potential interacting proteins of N-Myc from endogenous co-IPs, we conducted three N-Myc co-IP using N-Myc antibody and three corresponding immunoglobulin (IgG) co-IP using normal IgG antibody as negative control. To generate potential interacting proteins of N-Myc from exogenous co-IPs, we conducted three co-IPs using FLAG antibody or N-Myc antibody on 3xFLAG-N-Myc overexpressed 293T cell lysates, corresponding co-IP on 3xFLAG overexpressed 293T cell lysates using FLAG or N-Myc antibody, and co-IP on 3xFLAG-N-Myc overexpressed 293T cell lysates using IgG as negative control.

### Co-IP

SK-N-BE(2) (for endogenous co-IP and IP) and 293T cells overexpressing 3xFLAG-tagged N-Myc (for exogenous co-IP) were pretreated with 10 μg/ml MG132 (S2619; Selleck) for 6 h and lysed in co-IP cell lysis buffer (P0013J, Beyotime) with protease inhibitor (catalog no.: 05892970001; Roche) and phosphatase inhibitor (catalog no.: 04906837001; Roche) for suggested concentrations. To ensure the detection of acetylated N-Myc, we used 200 ng/ml trichostatin A (S1045; Selleck) for 12 h to increase the existed acetylation of N-Myc before cell harvest according to the literatures (19, 20). For endogenous N-Myc co-IP, N-Myc IP, and exogenous N-Myc co-IP using N-Myc antibody, cell lysates were incubated with N-Myc primary antibody overnight at 4 °C followed by 4 h at 4 °C with protein A/G magnetic beads (B23201; Bimake). For exogenous N-Myc co-IP using FLAG magnetic beads, cell lysates were incubated with anti-FLAG magnetic beads (B26102; Bimake) overnight at 4 °C. For endogenous and exogenous N-Myc co-IPs, magnetic beads were washed with IP wash buffer (50 mM Tris, 150 mM NaCl, 0.5% Tween-20, pH 7.5) three times after incubation. For N-Myc IP, magnetic beads were washed with IP wash buffer (50 mM Tris, 150 mM NaCl, 2% Tween-20, pH 7.5) at least four times after incubation. The bound proteins were eluted by boiling at 95 °C with 2% SDS for subsequent usage.

### HPLC–MS/MS Analysis

Protein products from co-IP were reduced with 5 mM dithiothreitol at 55 °C for 30 min, and Cys residues were alkylated with 15 mM iodoacetamide at room temperature in the dark for 30 min. Trypsin was added at an enzyme-to-substrate ratio of 1:100 (w/w), and the mixture was digested overnight at 37 °C with shaking (1500 r.p.m.). Samples were concentrated under vacuum and dissolved in a buffer containing 0.1% formic acid after desalting using a C8 StageTip according to the manufacturer's protocol. Four microliters of each sample were analyzed by UltiMate 3000 HPLC (Thermo Fisher Scientific) and a Q Exactive Plus mass spectrometer (Thermo Fisher Scientific), generating raw MS/MS spectra for each sample. MaxQuant 1.6.17.0 (Max-Planck-Institute of Biochemistry) and Perseus 1.6.15.0 (Max-Planck-Institute of Biochemistry) were used for protein identification, quantification, and statistical analysis. Raw MS/MS spectra were searched against the UniProt Knowledgebase for *Homo sapiens* (download date: May, 14, 2020; entries: 20,365). And intensity-based label-free quantification was used in protein quantification. The settings of protein quantification and identification using MaxQuant were as follows. Trypsin/P was set as the proteolytic enzyme with two missed or nonspecific cleavages permitted. Carbamidomethyl (C) was set as fixed modification and acetyl (K), oxidation (M), phospho (STY), and GlyGly (K) were set as variable modifications. The mass tolerance for precursor ions was set as 20 ppm in first search and 4.5 ppm in main search and that for fragment ions was set as 0.5 Da. The maximum false discovery rate for proteins and peptides was 0.01, and the minimum peptide length was seven amino acids.

### Proteomics Data Processing and Bioinformatic Analysis

After protein quantification using MaxQuant, potential N-Myc-interacting proteins were screened according to the following criteria (21). First, only proteins identified with at least two unique peptides were considered. Second, proteins to be considered as N-Myc-interacting proteins had to be identified only in the N-Myc co-IP groups (3xFLAG-N-Myc group in exogenous co-IP and N-Myc antibody group in endogenous co-IP) but not in the control groups (3xFLAG group in exogenous co-IP and IgG antibody group in endogenous co-IP) or had an enrichment ratio larger than 10 (N-Myc co-IP group/control group). Third, only proteins identified with intensity in two of three replicates from the exogenous and endogenous N-Myc co-IP groups were included.

GO and Kyoto Encyclopedia of Genes and Genomes (KEGG) analysis were carried out using STRING (https://www.string-db.org/) (22) with false discovery rate ≤0.01 and gene counts ≥10. The visualization of GO and KEGG analysis was conducted using RStudio (RStudio, Inc.).

### Synthetic peptides for validation

Peptides for validation of identified PTM residues of N-Myc were synthesized by KE BIOCHEM. One hundred nanograms of peptide was dissolved in a buffer containing 0.1% formic acid after desalting using a C8 StageTip according to the manufacturer's protocol. Modified peptide sequences were as follows: acetylated peptide AGAALPAELAHPAAECVDPAVVFPFPVNK(Ac)R and ubiquitinated peptide AGAALPAELAHPAAECVDPAVVFPFPVNK(gl)R.

### *In Vitro* Acetylation Assay

Recombinant glutathione-*S*-transferase (GST)-N-Myc protein (catalog no.: ABIN1311728; Antibodies-online) was incubated with recombinant p300 protein (catalog no.: 81158; Active Motif) with or without the p300 inhibitor CPI-637 (catalog no.: S8190; Selleck) in 50 mM Tris buffer (pH 8.0) containing 2% glycerol, 0.1 mM EDTA, 1 mM dithiothreitol, and acetyl-CoA for 1 h at 30 °C (23). Products were then prepared for MS analysis or boiled with SDS-PAGE protein loading buffer (catalog no.: 20315ES05; Yeasen) for Western blot analysis.

### *In Vitro* Ubiquitination Assay

Recombinant GST-N-Myc protein was incubated with or without recombinant p300 protein in a ubiquitination reaction containing 20 mM Hepes (pH 7.5, catalog no.: H3375; Sigma–Aldrich), 5 mM MgCl_2_ (catalog no.: AM9530G; Thermo Fisher Scientific), 2 mM dithiothreitol (catalog no.: R0861; Thermo Fisher Scientific), 2 mM ATP (catalog no.: A6559; Sigma–Aldrich), 5 mg of ubiquitin (catalog no.: U5507; Sigma–Aldrich), 20 mM MG132, and 5 μl/reaction crude rabbit reticulocyte lysate (catalog no.: L4151; Promega) for 1 h at 30 °C (24). Products were then subjected to IP using GST antibody (catalog no.: 2624; Cell Signaling Technology) and protein A/G magnetic beads before Western blot.

### siRNA Knock Down

Control siRNA and three different siRNAs specific for EP300 and NAT10, respectively, were chemically synthesized (RiboBio, China). Cells were seeded in 6-well plates at 30 to 40% confluency 24 h before transfection. RNAi transfections were performed using RNAiMAX (catalog no.: 13778150; Thermo Fisher Scientific) following the manufacturer's protocol. Cells were harvested for subsequent experiments 48 to 72 h after transfection.

Target sequences were as follows:

*EP300*-1: 5′-CGACTTACCAGATGAATTA-3′

*EP300*-2: 5′-GCACAAATGTCTAGTTCTT-3′

*EP300*-3: 5′-AGATGAGAGTTTAGGCCGC-3′

*NAT10*-1: 5′-GGAATATGGTGGACTATCA-3′

*NAT10*-2: 5′-GGACTGCTGTAAGACTCTA-3′

*NAT10*-3: 5′-GTACTCCAATATCTTTGTT-3′

*MYCN*: 5′-CTGAGCGATTCAGATGATGAA-3′

### Western Blot

Western blot was conducted as previously described (25). In brief, protein samples were loaded onto a polyacrylamide gel for electrophoresis and then electrotransferred onto polyvinylidene difluoride membranes (Bio-Rad). Blots were blocked with 5% bovine serum albumin at room temperature for 2 h and incubated with the selected primary antibodies at 4 °C overnight. After washing the membranes for three times, the membranes were incubated with second antibody for 1 h at room temperature. Bands were visualized by electrogenerated chemiluminescence (Pierce Biotechnology) with the Bio-Rad ChemiDoc XRS imaging system. Primary antibodies specific for p300 (catalog no.: 86377; 1:1000 dilution), N-Myc (catalog no.: 51705; 1:1000 dilution), and β-actin (catalog no.: 3700; 1:1000 dilution) were purchased from Cell Signaling Technology. NAT10 (catalog no.: ab194297; 1:1000 dilution) was purchased from Abcam. The results were quantitated using ImageJ (version 1.8.0, National Institutes of Health).

### RNA Isolation and Quantitative Real-Time PCR

Total RNA was isolated using TRIzol (catalog no.: T9108; Takara) and then reverse transcribed with the PrimeScript RT reagent Kit (catalog no.: RR037A; Takara). Quantitative real-time PCR was conducted using SYBR Green Master Mix (catalog no.: 11198ES03; Yeasen) and specific primers produced by GENEWIZ as follows.

*MYCN* forward: 5′-CGCAAAAGCCACCTCTCATTA-3′

*MYCN* reverse: 5′-TCCAGCAGATGCCACATAAGG-3′

*EP300* forward: 5′-TCCGAGACATCTTGAGACGACAG-3′

*EP300* reverse: 5′-GGGTTGCTGGAACTGGTTATGG-3′

*GAPDH* forward: 5′-CATGAGAAGTATGACAACAGCCT-3′

*GAPDH* reverse: 5′-AGTCCTTCCACGATACCAAAGT-3′

### Cell Counting, Colony-Formation Assay, and Cell Invasion Assay

Cells were seeded in a 96-well plate at 30 to 40% confluency, and cell viability was assessed on days 0, 2, 3, and 4 to generate a cell proliferation curve using a CCK8 Cell Counting Kit (catalog no.: 40203ES60; Yeasen) according to the manufacturer’s protocol. To determine the colony-formation capacity of cells under different treatments, 1000 SK-N-BE(2) and IMR-32 cells for each group were seeded in a 6-well plate and cultured until apparent colony formation. Colonies were stained with crystal violet (catalog no.: C0121; Beyotime Biotechnology), and the numbers of total colonies were counted and compared using ImageJ and GraphPad Prism 7 (GraphPad Software, Inc). Cell invasion assays were performed with an invasion chamber (catalog no.: 3422; Corning) pretreated with Matrigel added on top for 4 h in a 12-well plate. A total of 100,000 SK-N-BE(2) cells or 150,000 IMR-32 cells under different treatments were then washed with serum-free medium and seeded into the chamber. Cells were fixed and stained as previously described after incubation for 36 h at 37 °C (26). Five fields were randomly selected and photographed under a microscope in each chamber. The numbers of total cells in the chamber were counted and compared using ImageJ and GraphPad Prism 7.

### NB Dataset Analysis

For analysis of survival correlations, patient data were accessed through the R2: Genomics Analysis and Visualization Platform (http://r2.amc.nl). Overall survival analysis with *EP300* mRNA expression level in *MYCN*-amplified NB cases was assessed using patient data from Kocak (GSE45547) and SEQC (GSE49710) datasets (cutoff modus = scan, minimal group size = 8, and *MYCN* status = *MYCN*-amp).

### Statistical Analysis

Data analysis was carried out using SPSS software, version 20 (IBM Corporation) and GraphPad Prism 7 (GraphPad Software, USA). The results were presented as the mean  ±  SD. Differences were determined using a one-tailed or two-tailed paired Student’s *t* test or Mann–Whitney test as appropriate. A *p*  value ≤0.05 was considered statistically significant.

## Results

### The Proteomics Workflow for Identifying N-Myc-Interacting Proteins and PTM Residues

**Fig. 1:**
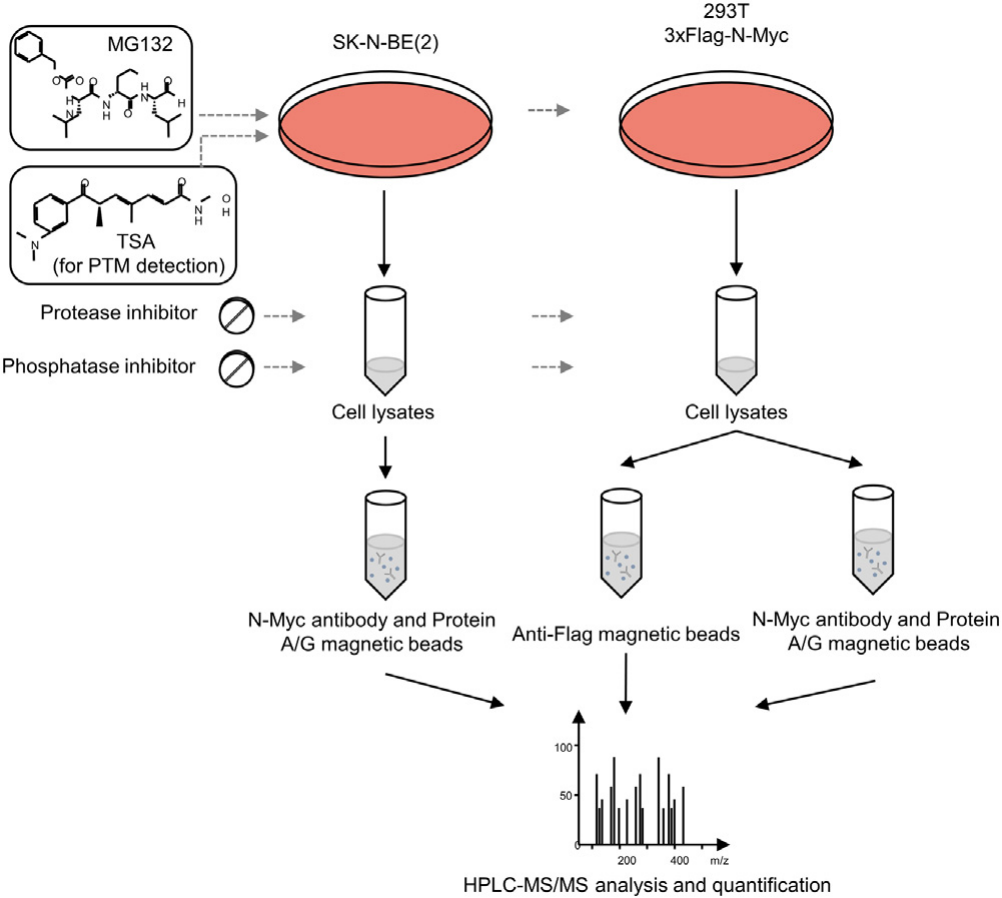
**Detecting PTMs of N-Myc- and N-Myc-interacting proteins by co-IP and HPLC–MS/MS.** Strategy of discovering PTMs of N-Myc and both endogenous and exogenous N-Myc-interacting proteins. co-IP, coimmunoprecipitation; PTM, post-translational modification.

**Supplementary Figure 1:**
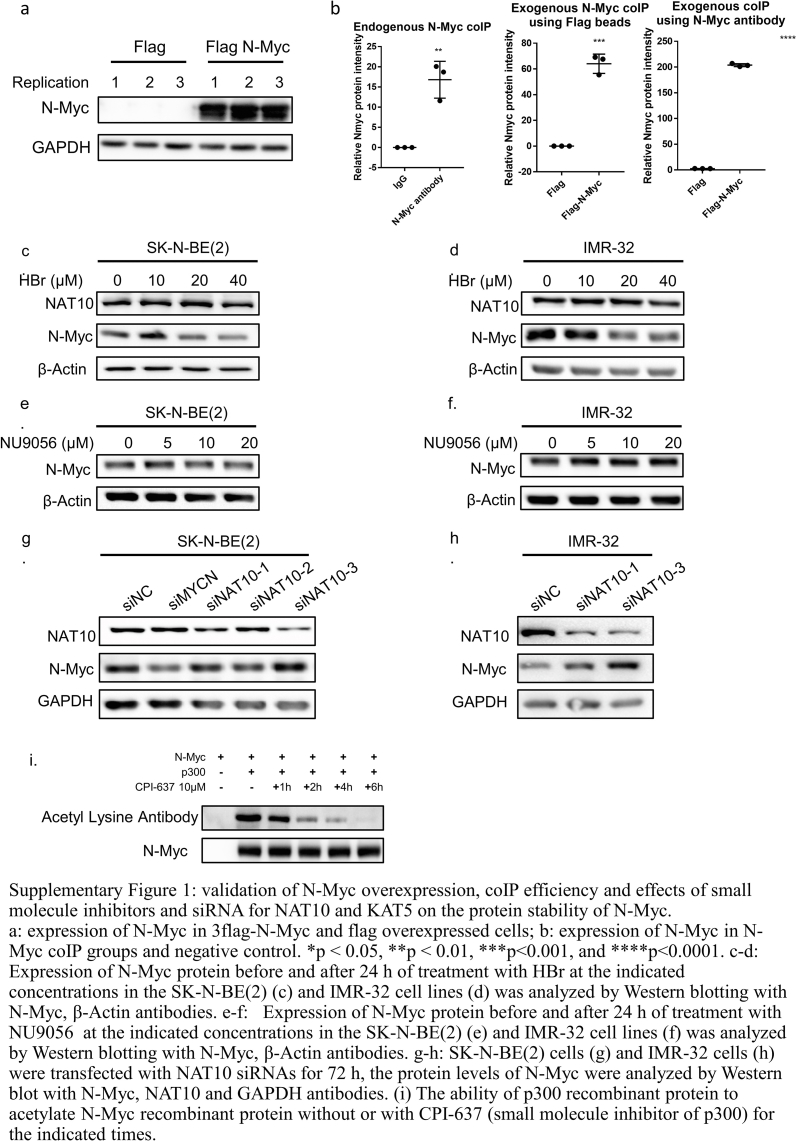


To identify the PTM residues and interacting proteins of N-Myc (both endogenous and exogenous), a quantitative proteomics strategy was employed by combining lentiviral infection, co-IP, and HPLC–MS/MS analysis. We constructed 293T cells with stable overexpression of 3xFLAG-N-Myc and 3xFLAG using a lentivirus-mediated overexpression system (supplemental Fig. S1*A*). As shown in Figure 1, SK-N-BE(2) cells and 293T cells expressing 3xFLAG-N-Myc used for N-Myc IP. For endogenous co-IP and N-Myc IP, SK-N-BE(2) cell lysates were treated with protein A/G magnetic beads and N-Myc antibody or IgG as a negative control. For exogenous co-IP using FLAG magnetic beads, 293T cells expressing 3xFLAG-N-Myc were lysed and treated with anti-FLAG magnetic beads, and 293T cells expressing 3xFLAG were lysed as a negative control. For exogenous co-IP using N-Myc antibody, 293T cells expressing 3xFLAG-N-Myc lysed and treated with IgG and 293T cells expressing 3xFLAG lysed and treated with N-Myc antibody were used as negative controls. Co-IP samples were then pretreated and subjected to HPLC–MS/MS analysis. The raw data of four N-Myc IP, six endogenous N-Myc co-IP, and 15 exogenous N-Myc co-IP were generated and submitted to a public repository (https://www.iprox.cn/).

### Identification of PTM Residues of N-Myc

**Fig. 2:**
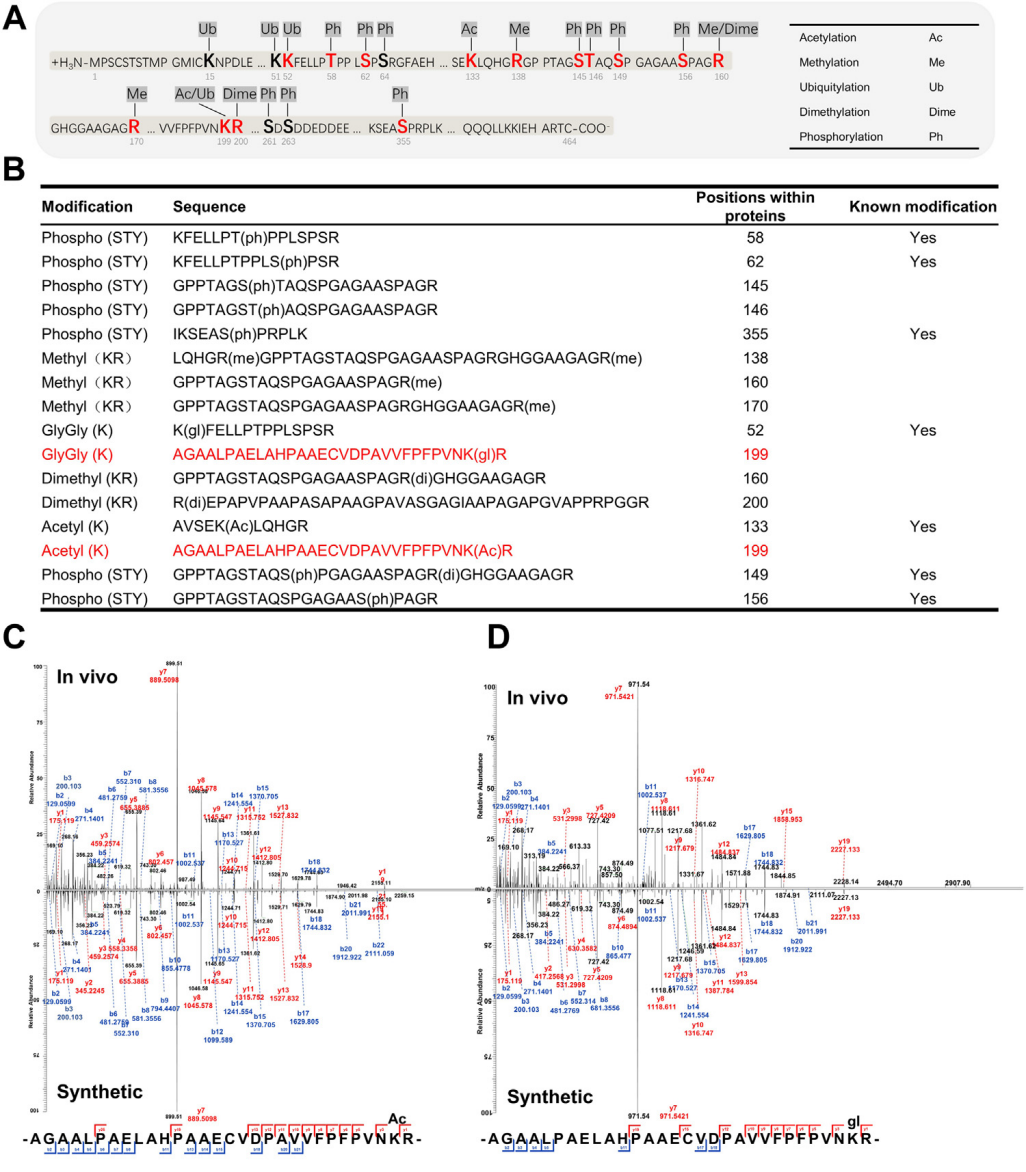
**PTMs of N-Myc detected by IP and HPLC–MS/MS and synthetic peptide validation of the indicated peptides and modification residues.***A*, distribution of PTM residues of N-Myc detected by IP and HPLC–MS/MS on its amino acid sequence from the N terminus to the C terminus (1–464). The abbreviations of the mentioned PTM types are listed on the *right*. *B*, the PTM type, peptide sequence, and PTM residues of each identified PTM of N-Myc are shown in the table. Whether it is a known modification is also indicated in the *rightmost column*. *C*, mass spectra of peptide (AGAALPAELAHPAAECVDPAVVFPFPVNK(Ac)R) with acetylation on lysine 199 of N-Myc detected in the N-Myc IP sample (*upper panel*) and the synthetic peptide (*lower panel*). *D*, mass spectra of peptide (AGAALPAELAHPAAECVDPAVVFPFPVNK(Ac)R) with ubiquitylation on lysine 199 of N-Myc detected in the N-Myc IP sample (*upper panel*) and the synthetic peptide (*lower panel*). IP, immunoprecipitation; PTM, post-translational modification.

**Supplementary Figure 2:**
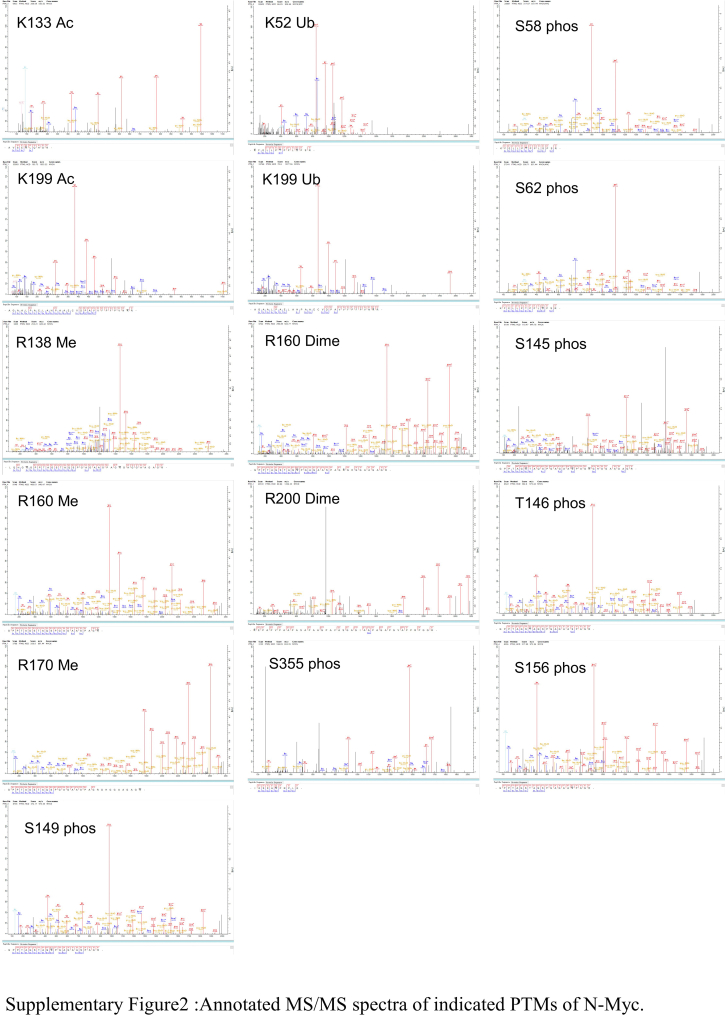


Residues of PTMs of N-Myc were generated from four N-Myc IP tandem mass spectrometry (MS) data using MaxQuant. We detected 16 PTM residues from replicates of four N-Myc IP results, including acetylation, methylation, ubiquitylation, demethylation, and phosphorylation. The detailed location information of these modifications within each peptide sequence is listed in supplemental Table S1, and the spectra of these modifications are shown in supplemental Fig. S2. The BioGRID database (https://theBioGRID.org/), PhosphoSitePlus (https://www.phosphosite.org/) database, and iPTMnet database (https://research.bioinformatics.udel.edu/iptmnet/) together listed 21 PTM residues of N-Myc, and seven of them overlapped with our findings (Fig. 2, *A* and *B* and supplemental Table S2). Notably, two different PTM types existed on the same amino acid residue: acetylation and ubiquitylation on lysine 199 of N-Myc.

To validate the existence of acetylation and ubiquitylation on lysine-199, peptides between residues 171 and 200 of N-Myc containing acetylation or ubiquitylation on lysine-199 were artificially synthesized and subjected to HPLC–MS/MS analysis. The raw data of these samples were submitted to a public repository (https://www.iprox.cn/), and the detailed location information of these modifications within each peptide sequence are listed in supplemental Table S3. As shown in Figure 2, *C* and *D*, the mass spectra of synthesized peptides showed a peak pattern identical to the peak pattern of our N-Myc co-IP results, confirming the acetylation and ubiquitylation of lysine-199 of N-Myc. The raw data of two synthesized peptide samples were submitted to a public repository (https://www.iprox.cn/).

### Identification of the N-Myc-Interacting Proteins Using Proteomics

**Fig. 3:**
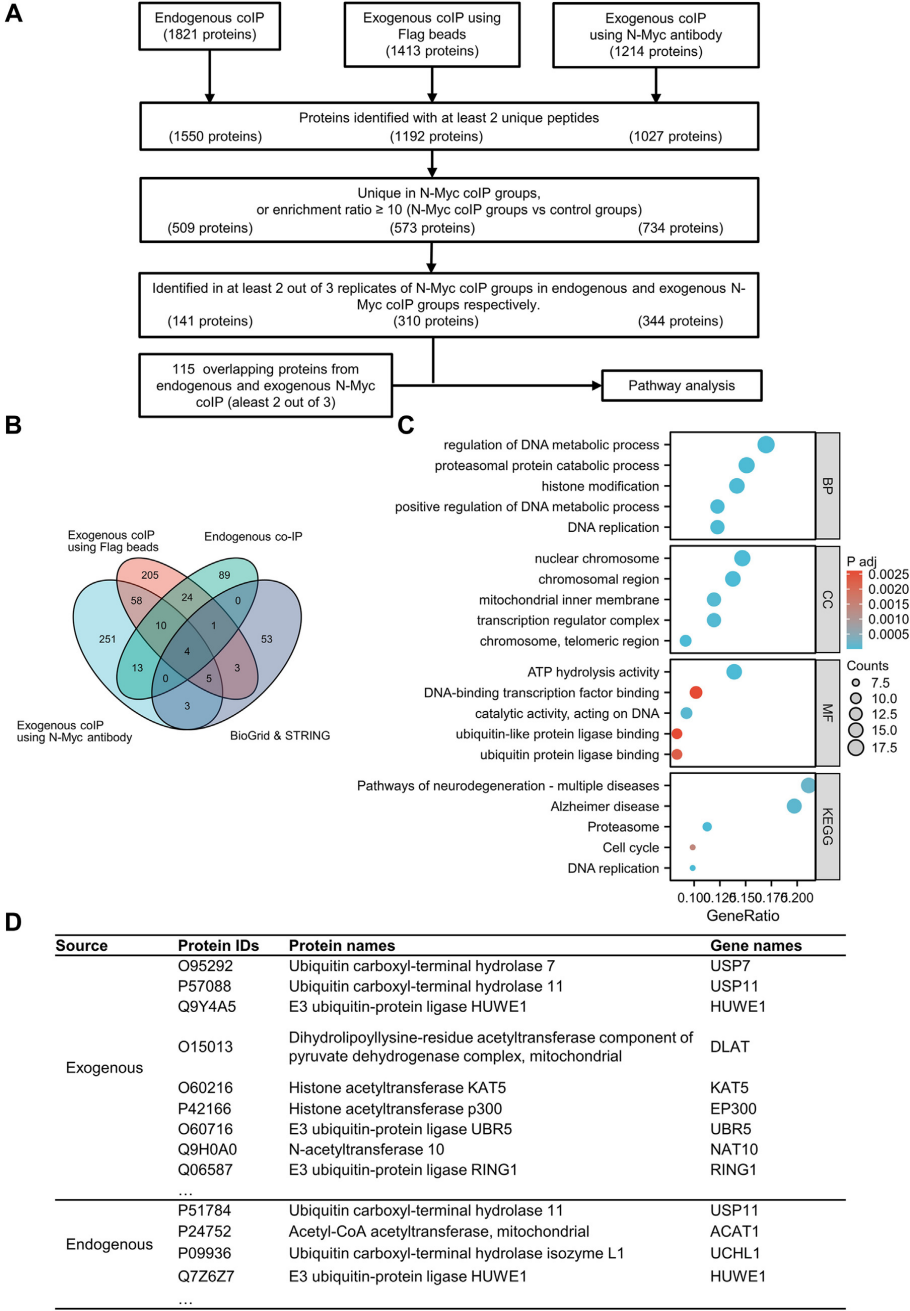
**The profiles of protein expression in the control and N-Myc co-IP groups of endogenous and exogenous N-Myc-co-IP experiments.***A*, screening criteria used to identify potential N-Myc-interacting proteins and corresponding results in three independent co-IP experiments. *B*, Venn diagram of previously identified N-Myc-interacting proteins from the BioGRID and STRING databases (*lower right*), endogenous (*upper right*), and two exogenous (*upper and lower left*) N-Myc-interacting proteins we discovered. *C*, top five enriched biological process (BP), cellular component (CC), and molecular function (MF) terms, and top five enriched Kyoto Encylopedia of Genes and Genomes (KEGG) pathways for 39 potential N-Myc-interacting proteins. *D*, list of several acetylation and ubiquitination-related proteins significantly enriched in N-Myc endogenous and exogenous co-IP experiments. Co-IP, coimmunoprecipitation.

To identify enzymes that potentially regulate the PTMs of N-Myc, N-Myc-interacting proteins were analyzed from endogenous and exogenous N-Myc co-IP LC–MS/MS results using the quantitative proteomics strategy described previously. The detailed information of protein identification and quantification results of three independent co-IP experiments are listed in supplemental Table S4. Both endogenous and exogenous N-Myc co-IPs were conducted three times, and quantification of N-Myc after co-IP and LC–MS/MS analysis was shown in supplemental Fig. S1*B*. The screen criteria and results demonstrated in Figure 3*A* showed that 1550, 1192, and 1027 proteins were identified with two or more unique peptides in endogenous and two exogenous N-Myc co-IP, respectively. After enrichment ratio calculation and screening following the procedure described previously, there were 141, 310, and 344 enriched proteins in endogenous and two exogenous N-Myc co-IP, respectively (supplemental Table S5). Thus far, only 69 N-Myc-interacting proteins have been reported and validated in the literature according to the BioGRID and STRING databases (supplemental Table S6). Figure 3*B* demonstrating the overlap between these four lists showed that 115 proteins (including N-Myc) were identified in at least two of the three co-IP experiments in our study, and 14 among them (including N-Myc) fit the screening standard in all three independent co-IP experiments (Table 1). Besides, several previously known N-Myc-interacting proteins were also identified as potential interactor of N-Myc in our results, including MAX, HUWE1, and TRRAP. Even though we used three different datasets to screen potential interactors of N-Myc, the overlapping lists may still contain some nonspecific interactors without experiment validation.

**Table 1:** The quantitative information of 14 overlapping proteins from three co-IP experiments

Overlap proteins	Endogenous co-IP			Exogenous co-IP using FLAG beads			Exogenous co-IP using N-Myc antibody			
	Ratio_1	Ratio_2	Ratio_3	Ratio_1	Ratio_2	Ratio_3	Ratio_1	Ratio_2	Ratio_3	
SLC25A12	Unique	Unique	0	50.93	Unique	Unique	Unique	Unique	Unique	
PSMA1	Unique	Unique	0	0	Unique	Unique	Unique	Unique	Unique	
PSMC2	Unique	Unique	0	Unique	Unique	6.71	Unique	4.85	Unique	
PSMC1	Unique	Unique	0	Unique	Unique	0.94	Unique	Unique	Unique	
HSPH1	Unique	Unique	2.68	Unique	Unique	Unique	Unique	Unique	Unique	
HCFC1	14.29	20.96	Unique	Unique	Unique	59.27	Unique	Unique	Unique	
TRRAP	Unique	44.45	Unique	Unique	Unique	Unique	Unique	Unique	Unique	
MYCN	Unique	1033.17	Unique	Unique	6033.54	3465.14	Unique	Unique	Unique	
EP400	0	Unique	Unique	Unique	Unique	Unique	Unique	Unique	Unique	
SLC25A10	0	Unique	Unique	Unique	Unique	Unique	Unique	Unique	Unique	
SMC2	Unique	Unique	Unique	Unique	Unique	9.23	Unique	Unique	Unique	
MAX	Unique	Unique	Unique	Unique	Unique	Unique	Unique	Unique	Unique	
PYCRL	Unique	Unique	Unique	Unique	Unique	Unique	Unique	Unique	Unique	
HUWE1	Unique	Unique	Unique	Unique	Unique	Unique	Unique	Unique	Unique	

Unique: Unique in N-Myc co-IP groups.

### Bioinformatic Analysis of Potential N-Myc-Interacting Proteins

To further understand the function of the identified N-Myc-interacting proteins, the STRING server (http://www.string-db.org/) was used to search for significantly enriched GO terms and KEGG pathways of 115 proteins identified in two of three endogenous or exogenous N-Myc co-IPs. The enriched biological processes (BPs), molecular functions (MFs), cellular components (CCs), and KEGG pathways of N-Myc-interacting proteins are summarized in supplemental Table S7. The top five of each category are listed in Figure 3*C*. Based on KEGG pathway enrichment, there were several enriched pathways related to neurological diseases and cell cycle, such as Alzheimer’s disease, and pathways of neurodegeneration. Besides, KEGG pathways associated with protein degradation including proteasome was also enriched. The enriched GO terms were related to various aspects of DNA binding, chromatin, and protein binding, such as DNA-binding transcription factor binding (MF), nuclear chromosome (CC), transcription regulator complex (CC), and DNA replication (BP). All the GO terms mentioned previously were more or less related to the function of N-Myc itself, but some of the enriched GO terms might have an impact on the regulation of N-Myc proteins, such as histone modification (BP) and ubiquitin protein ligase binding (MF). Several proteins identified in our N-Myc-interacting protein lists were related to the regulation of N-Myc protein modification and degradation and may play a role in regulating PTMs.

### P300 Regulated the Expression of N-Myc in *MYCN*-Amplified NB Cells

**Fig. 4:**
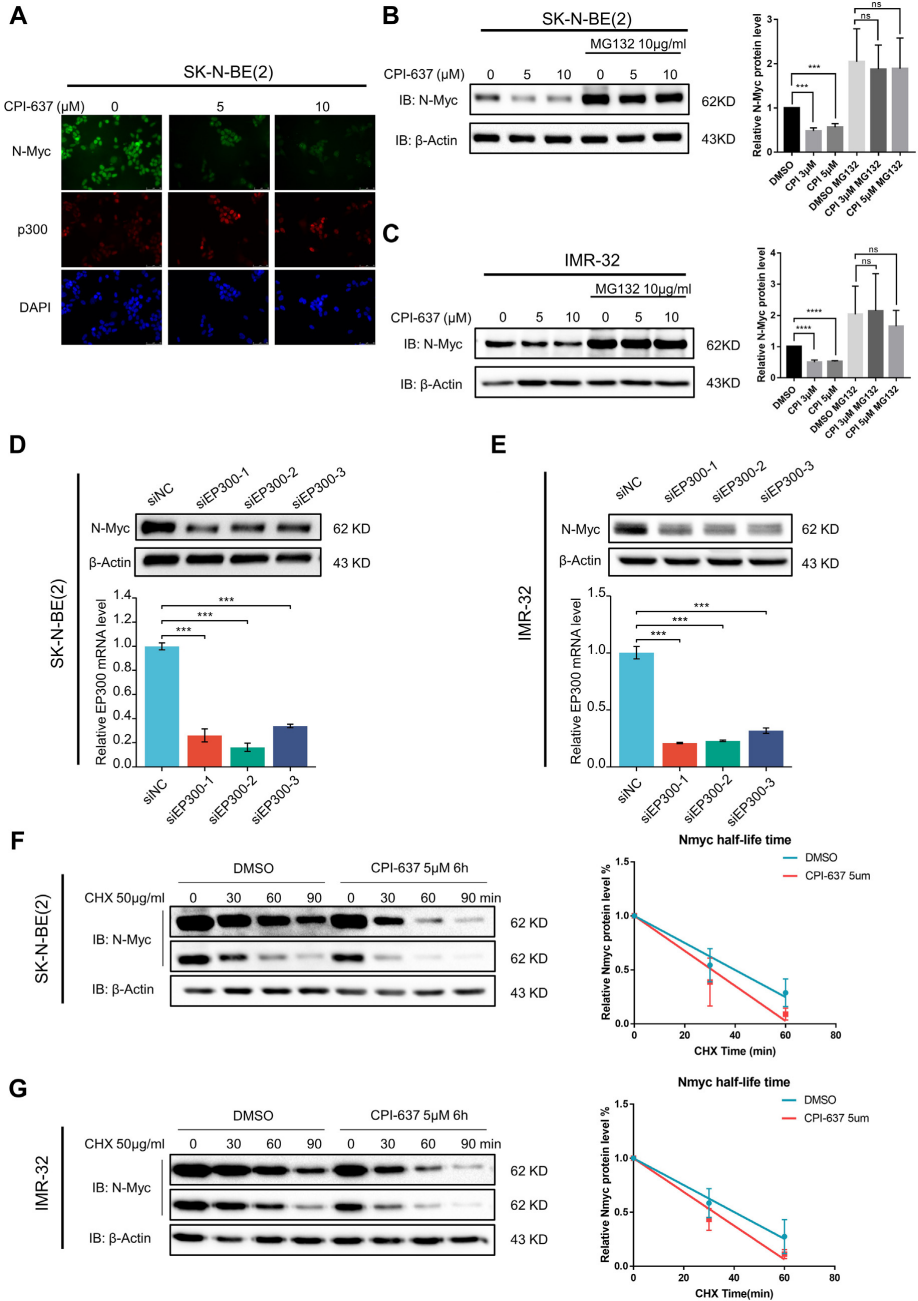
**A p300 small-molecule inhibitor downregulated N-Myc by reducing its protein stability in *MYCN*-amplified neuroblastoma cell lines.***A*, immunofluorescence of p300 and N-Myc proteins in SK-N-BE(2) cells after 6 h of treatment with CPI-637 at the indicated concentrations. *B* and *C*, expression of N-Myc protein before and after 6 h of treatment with CPI-637 at the indicated concentrations with or without MG132 (10 μg/ml) in the SK-N-BE(2) (*B*) and IMR-32 cell lines (*C*) was analyzed by Western blot with N-Myc and β-actin antibodies. Quantification was conducted using ImageJ and Prism 7. ∗*p* < 0.05, ∗∗*p* < 0.01, ∗∗∗*p* < 0.001, and ∗∗∗∗*p* < 0.0001. *D* and *E*, SK-N-BE(2) cells (*D*) and IMR-32 cells (*E*) were transfected with *EP300* siRNAs for 72 h. The protein levels of N-Myc were analyzed by Western blot with N-Myc and β-actin antibodies (*upper panel*), and the knockdown efficiencies of *EP300* siRNAs were analyzed by quantitative PCR with *EP300* and *GAPDH* primers (*lower panel*). ∗*p* < 0.05, ∗∗*p* < 0.01, and ∗∗∗*p* < 0.001. *F* and *G*, SK-N-BE(2) cells (*G*) and IMR-32 cells (*F*) were pretreated with CPI-637 (5 μM) for 6 h and then treated with cycloheximide (CHX) (50 μg/ml) for the indicated times. The expression of N-Myc was analyzed by Western blot with the indicated antibodies (*left panel*). The half-life of the N-Myc protein was analyzed by linear regression using GraphPad (*right panel*).

**Fig. 5:**
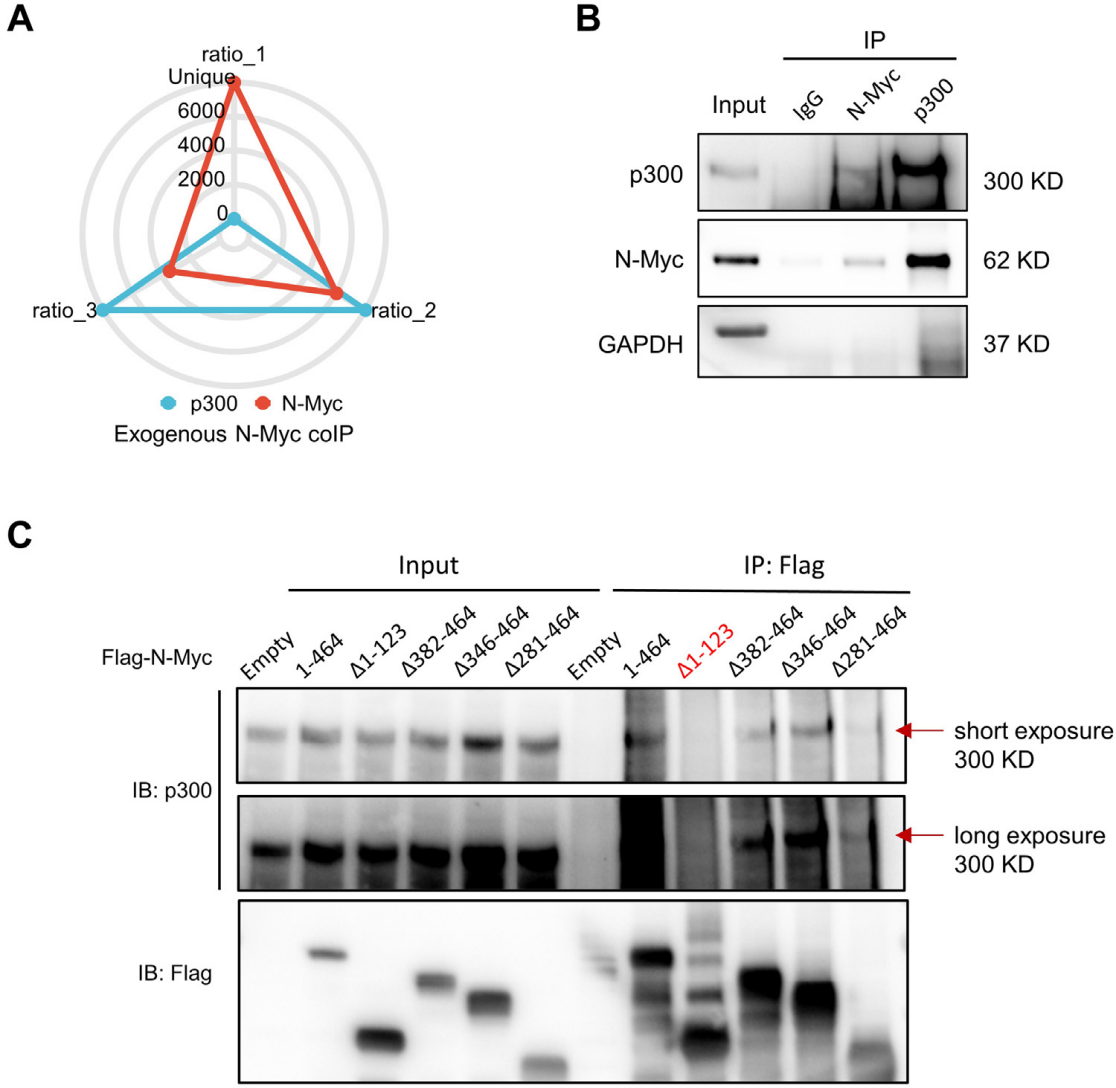
**p300 interacted with full-length and different deletion mutant N-Myc protein and colocalized in the neuroblastoma cell line SK-N-BE(2).***A*, the radar chart shows the expression ratio of N-Myc and p300 between exogenous 3xFLAG-N-Myc immunoprecipitation (IP) and 3xFLAG IP. Expression ratios range from 0 to 6000, “unique” labeled on the most outer ring of the radar chart means only identified in 3xFLAG-N-Myc IP group. *B*, the co-IP of endogenous N-Myc or p300 was conducted using N-Myc or p300 antibody in SK-N-BE(2) cell lysates. The N-Myc and p300 expression in co-IP products was detected by Western blot using N-Myc and p300 antibody. Normal immunoglobulin G (IgG) antibody co-IP was used as a negative control. *C*, the co-IP of full-length and different deletion mutant N-Myc protein with FLAG tag were conducted using FLAG magnetic beads. The expression of N-Myc and p300 in different input and co-IP protein products was analyzed by Western blot. The band of p300 was presented under both short exposure (*upper band*) and long exposure (*lower band*) condition.

In the HPLC–MS/MS results of endogenous and exogenous N-Myc co-IP, several acetylation-related proteins were significantly enriched in endogenous and exogenous co-IP groups (Fig. 3*D*). We tested the effect of small-molecule inhibitors for three different acetylation-related proteins, including p300, KAT5, and NAT10, on N-Myc protein levels in *MYCN*-amplified NB cells. As a result, both p300 inhibitor (CPI-637) and NAT10 inhibitor (Remodelin hydrobromide), but not KAT5 inhibitor (NU9056), reduced N-Myc protein expression in *MYCN*-amplified NB cells (Figs. 4, *A*–*C* and S1, *C* and *F*). To avoid possible off-target effects of inhibitors of p300 and NAT10, we ablated p300 and NAT10 using siRNA in SK-N-BE(2) and IMR-32 cells, respectively. P300 downregulation using specific siRNAs reduced N-Myc expression levels in *MYCN*-amplified NB cells, but NAT10 siRNAs failed to do so (Figs. 4, *D* and *E* and S1, *G* and *H*). Furthermore, as shown in Figure 4, *B* and *C*, the N-Myc protein level was dramatically increased by treatment with MG132, and the downregulation effect on N-Myc of CPI-637 was reversed when the proteasomal degradation pathway was blocked by MG132. Moreover, the protein half-life of N-Myc was shortened after treatment with CPI-637 for 6 h in both SK-N-BE(2) and IMR-32 cells (Fig. 4, *F* and *G*). These results indicated that CPI-637 downregulated N-Myc by increasing its degradation *via* the proteasome pathway and possibly by altering its ubiquitination level. In addition, p300 was identified as a potential N-Myc-interacting protein in exogenous N-Myc co-IP (Fig. 5*A*).

### P300 Interacted With Endogenous N-Myc, Full-Length FLAG-N-Myc, and Amino Acid 1 to 123 Reserved Deletion Mutant N-Myc

Based on former results, we used cell lysates of SK-N-BE(2) to perform endogenous co-IP using normal IgG, N-Myc, and p300 antibody to validate the interaction between N-Myc and p300. Our results demonstrated that endogenous N-Myc was detected in co-IP products using p300 antibody, and p300 was detected in co-IP products using N-Myc antibody. Both N-Myc and p300 was not detected from normal IgG co-IP by the same approach (Fig. 5*B*). We next constructed plasmids that overexpressed full length and four deletion mutated N-Myc with a FLAG tag (Δ1–0123, Δ382–464, Δ346–464, and Δ281–464) to validate exogenous interactions between N-Myc and p300 and to detect the crucial region of N-Myc for binding with p300. About 72 h after transfection, the total proteins of six corresponding 293 cells were extracted and subjected to co-IP using FLAG magnetic beads. Western blot using FLAG antibody of the input and co-IP protein products confirmed the success of N-Myc overexpression in 293 cells and the following co-IP (Fig. 5*C*). As a result, p300 was detected in all input 293 cell lysates (293 overexpressed empty vector, full-length N-Myc, and four deletion mutant N-Myc), and co-IP protein products of 293 cells overexpressed full-length, Δ382 to 464, Δ346 to 464, and Δ281 to 464 mutant N-Myc but not in 293 cells overexpressed empty and Δ1–123 N-Myc (Fig. 5*C*). This result demonstrated that p300 can interact with N-Myc, and the binding site of their interaction was probably within amino acid 1 to 123 of N-Myc protein.

### P300 Regulated the Post-Translational Acetylation and Ubiquitylation of N-Myc *In Vitro*

**Fig. 6:**
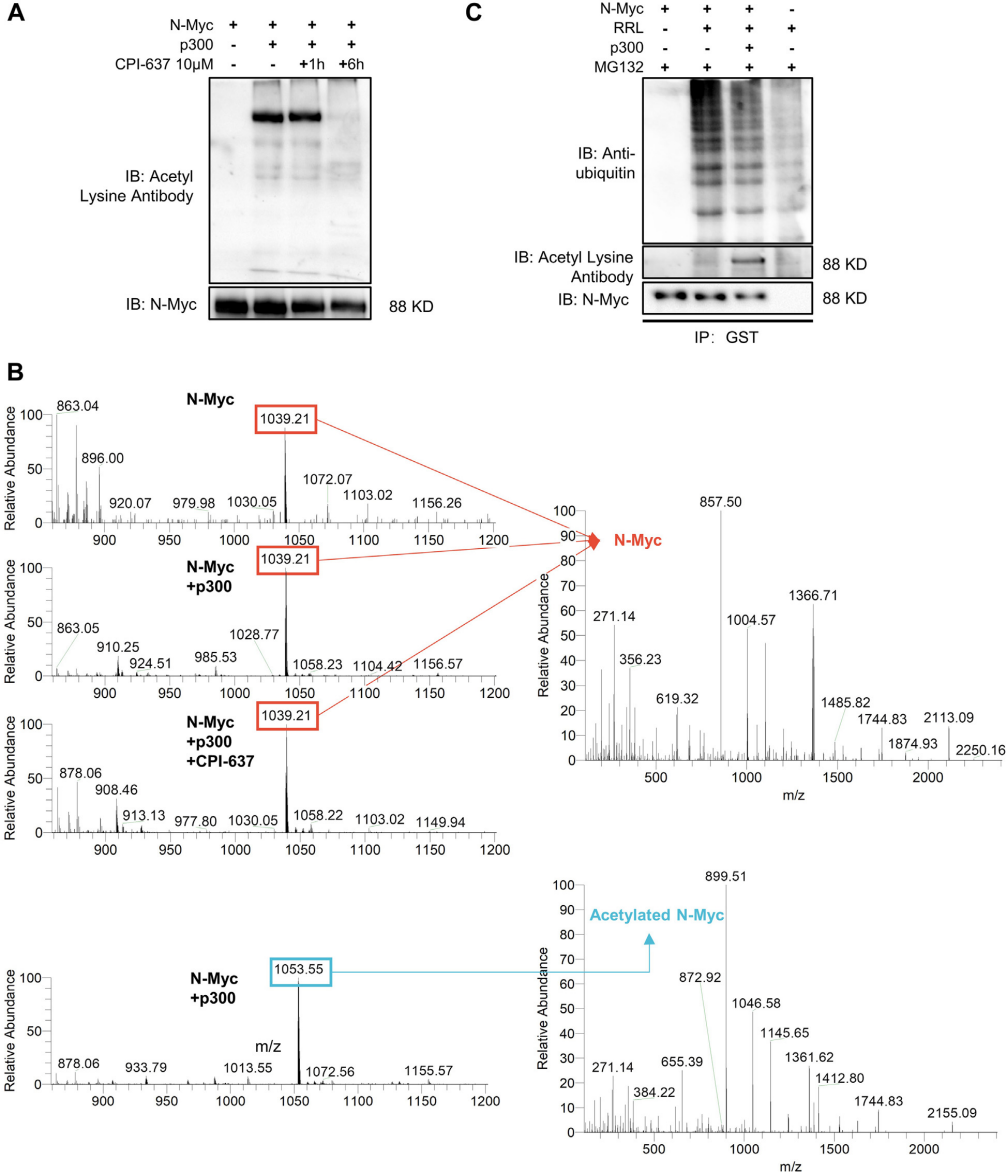
**p300 regulated both acetylation and ubiquitination of lysine-199 of N-Myc *in vitro*.***A* and *B*, acetylation of N-Myc could be modified by p300 *in vitro*. The ability of p300 recombinant protein to acetylate N-Myc recombinant protein without or with CPI-637 (small-molecule inhibitor of p300) for the indicated times was analyzed by Western blot with the indicated antibodies (*A*) and HPLC–MS/MS (*B*) after an *in vitro* acetylation assay. Peptide AGAALPAELAHPAAECVDPAVVFPFPVNKR of unmodified N-Myc (*m/z* = 1039.21) was identified in all products after three *in vitro* acetylation assays (*upper panel* of Fig. 6*B*), but acetylated N-Myc peptide (AGAALPAELAHPAAECVDPAVVFPFPVNK(Ac)R) (*m/z* = 1053.55) was identified only in N-Myc incubated with p300 (*lower panel* of *B*), referring to lane 2 in *A*. *Left panel* of *B*: mass spectrums of N-Myc or acetylated N-Myc peptide. *Right panel* of *B*, secondary mass spectrums of N-Myc or acetylated N-Myc peptide. *C*, N-Myc recombinant protein was incubated with or without p300 recombinant protein in an *in vitro* ubiquitination and acetylation system (rabbit reticulocyte lysate [RRL[) in the presence of MG132 (10 μg/ml), and then the acetylation levels and ubiquitination levels of N-Myc and N-Myc protein levels were tested by Western blot with N-Myc, antiubiquitin, and acetyl lysine antibodies.

As mentioned previously, p300 was a potential N-Myc-interacting protein resulting from exogeneous N-Myc co-IP and regulated N-Myc expression by altering its protein stability. We then performed *in vitro* acetylation and ubiquitylation assays to confirm the PTM regulatory effect of p300 on N-Myc. Western blot analysis of the *in vitro* acetylation of N-Myc under the indicated conditions revealed that the p300 recombinant protein acetylated N-Myc recombinant protein without or with CPI-637 coincubation for 1 h. However, when p300 recombinant protein was incubated with CPI-637 for 6 h, its ability to acetylate N-Myc was inhibited (Figs. 6*A* and S1*I*). The products of the *in vitro* acetylation assay were also processed by HPLC–MS/MS. The raw data of these samples were submitted to a public repository (https://www.iprox.cn/), and the detailed location information of these modifications within each peptide sequence are listed in supplemental Table S8. Peptide AGAALPAELAHPAAECVDPAVVFPFPVNKR representing amino acids 170 to 200 of the unmodified N-Myc protein were identified in all testing samples, including N-Myc only, N-Myc with p300, and N-Myc with both p300 and CPI-637. However, peptide AGAALPAELAHPAAECVDPAVVFPFPVNK(Ac)R with acetylation on lysine-199 was only identified in the sample that had both N-Myc and p300 in the reaction system of the *in vitro* acetylation assay (Fig. 6*B*). These results indicated that p300 can modify the post-translational acetylation on lysine-199 of the N-Myc protein *in vitro*, but we still had to verify whether the acetylation level of N-Myc affected its ubiquitylation level and therefore its protein stability. Thus, an *in vitro* ubiquitylation assay was carried out and subjected to Western blot. As a result, N-Myc itself was ubiquitylated in the rabbit reticulocyte lysate system without being acetylated (Fig. 6*C*, lane 2). However, in the presence of p300 recombinant protein, N-Myc was acetylated and presented with a lower level of ubiquitylation (Fig. 6*C*, lane 3). Taken together, these results showed that p300 can acetylate N-Myc and regulate its ubiquitylation level at the same time, resulting in regulation of the protein stability and protein level of N-Myc.

### P300 Played an Important Role in the Tumor Characteristics of *MYCN*-Amplified NB Cells

**Fig. 7:**
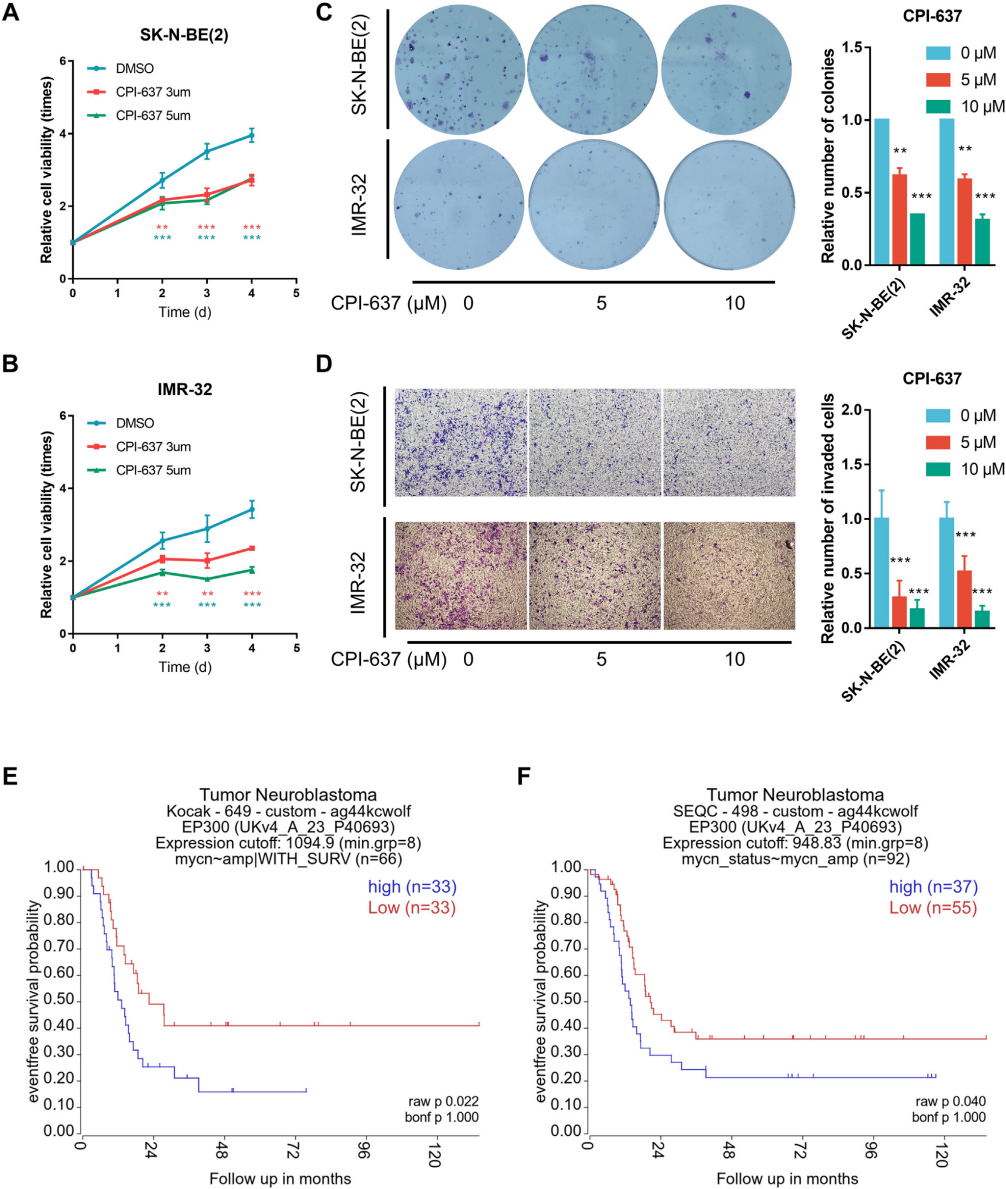
**p300 small-molecule inhibitor inhibited *MYCN*-amplified neuroblastoma cell proliferation, colony formation, and invasion.***A* and *B*, growth curves of SK-N-BE(2) cells (*A*) and IMR-32 cells (*B*) over 4 days without or with CPI-637 at the indicated concentrations. Cell viability was measured using a CCell Counting Kit-8 (CCK-8) assay on days 0, 2, 3, and 4. ∗*p* < 0.05, ∗∗*p* < 0.01, and ∗∗∗*p* < 0.001. *C*, colony-formation assay results of SK-N-BE(2) cells (*upper panel*) and IMR-32 cells (*lower panel*) over 14 days without or with CPI-637 at the indicated concentrations. The numbers of colonies among groups were counted and compared using GraphPad Prism 7. ∗*p* < 0.05, ∗∗*p* < 0.01, and ∗∗∗*p* < 0.001. *D*, transwell invasion assay results of SK-N-BE(2) cells (*upper panel*) and IMR-32 cells (*lower panel*) over 24 h without or with CPI-637 at the indicated concentrations. The numbers of invaded cells among groups were counted and compared using GraphPad Prism 7. ∗*p* < 0.05, ∗∗*p* < 0.01, and ∗∗∗*p* < 0.001. *E* and *F*, Kaplan–Meier analysis of event-free survival in *MYCN*-amplified neuroblastoma patients from the Kocak dataset (*E*, n = 66) and the SEQC dataset (*F*, n = 92) based on p300 expression with the log-rank test *p* value indicated, respectively.

Up to this point, we demonstrated that p300 regulated the N-Myc protein by modifying its PTMs. To evaluate the effect of p300 on the biological process of *MYCN*-amplified NB cells, we drew the cell growth curves of two *MYCN*-amplified NB cell lines under CPI-637 treatment at the concentration of 0, 3, and 5 μM. As shown in Figure 7, *A* and *B*, CPI-637 inhibited the proliferation of both SK-N-BE(2) and IMR-32 cells. Furthermore, CPI-637 can also reduce the colony-formation ability and invasion ability resulting from colony-formation assay (Fig. 7*C*) and cell-invasion assay (Fig. 7*D*) in *MYCN*-amplified NB cells. Thus, the inhibition of p300 in *MYCN*-amplified NBs is associated with a less malignant phenotype, less aggressive tumor behavior, and lower protein levels of N-Myc. Next, we applied CPI-637 on N-Myc knockdown *MYCN*-amplified NB cells. And results showed that proliferation of all groups was inhibited because of N-Myc knockdown, and the inhibition of CPI-637 did not further slowdown the cell proliferation, which further supported the theory that p300 modulated the proliferation of *MYCN*-amplified NB cells *via* regulating N-Myc expression (supplemental Fig. S3, *A*–*D*). Also, p300 has been reported to regulate the acetylation and expression of c-Myc in 293T cells, while our results showed slight regulatory effect of p300 inhibition or knockdown on c-Myc expression (supplemental Fig. S3, *E* and *F*), possibly because of diverse diseases and cell lines. Furthermore, we used two public datasets to assess the function of p300 in the pathogenesis of *MYCN*-amplified NB. Kaplan–Meier survival analysis of p300 in the population of *MYCN*-amplified NB in both datasets suggested that a higher level of p300 was correlated with lower event-free survival rates (Fig. 7, *E* and *F*).

## Discussion

*MYCN*, as an independent risk factor for poor prognosis in NB patients, is a member of the MYC family, a transcription factor with a basic helix–loop–helix domain, and a proto-oncogene (27). Therefore, targeting *MYCN* has become an important direction for the study of new treatment options for NB (28). The protein product N-Myc is almost entirely composed of α-helical structures and does not have a surface for ligand binding, making it difficult to directly use N-Myc as a drug target for the treatment of NB (6). As a result, there is currently no effective specific inhibitor directly targeting N-Myc. At this stage, research on targeting N-Myc was mainly focused on targeting aspects including DNA binding function, transcription, synthetic lethal interaction, oncogenic stabilization, and function of *MYCN* (29, 30).

On the one hand, the half-life of N-Myc is only 20 to 30 min, but N-Myc can be stably maintained at a high protein level in *MYCN*-amplified NB cells. On the other hand, there were studies showing that the high protein level of N-Myc may still appear in the nucleus of NB cells even with low level of *MYCN* mRNA and is closely related to the poor prognosis. The aforementioned results suggested that regulation of N-Myc at the protein level has an important influence on the abundance or activity of N-Myc protein in NB cells regardless of the amplification status of N-Myc at the mRNA level, providing an idea for selective intervention of N-Myc highly expressed NB with or without *MYCN* amplification (5).

The regulation of PTMs has a significant impact on the regulation of protein abundance after transcription. The four previously known interactors of N-Myc identified in all three independent experiments were closely related to the transcriptional function of N-Myc or its regulation. Among them, MAX (31) was a member of the basic helix–loop–helix leucine zipper and functioned as a transcriptional activator by forming a DNA-binding protein complex with MYC or MAD. TRRAP (32) belonged to PIK-related kinase family and played a vital role in MYC transcription activation and cell transformation regulated by MYC. HUWE1 (11) was a E3 ubiquitin-protein ligase that mediates ubiquitination and subsequent proteasomal degradation of target proteins, including N-Myc. EP400 (32) was a component of the NuA4 histone acetyltransferase complex and was required for transcriptional activation of E2F1 and MYC target genes during cellular proliferation. To date, 21 PTM residues of N-Myc have been reported and validated according to the BioGRID, PhosphoSitePlus, and iPTMnet databases (33), including several phosphorylation sites and ubiquitination sites. The phosphorylation of N-Myc has been well studied and proven to influence the protein stability of N-Myc (34). The protein kinase Gsk3 can phosphorylate N-Myc on threonine-58 to form a phosphodegron and subsequently initiate the ubiquitination and degradation of N-Myc *via* proteosomes mediated by Fbxw7 (35). Several strategies indirectly targeting the stability of N-Myc derived from this PTM regulatory mechanism, such as a ROCK2 small-molecule inhibitor, can activate the phosphorylation ability of Gsk3β on threonine-58 of N-Myc and therefore reinforce the degradation of N-Myc conducted by Fbxw7, resulting in downregulating N-Myc protein levels and suppressing the growth of *MYCN*-amplified NB cells *in vivo* (36). As demonstrated preiously, we identified 16 PTM residues by combining co-IP and HPLC–MS/MS, six of which were known PTM residues validated by studies, such as phosphorylation on threonine-58, serine-62, and serine-64. In addition, the novel PTM residues indicated in our study may provide new directions for indirect targeting of the N-Myc protein.

Among all these PTM residues, we noticed that both acetylation and ubiquitylation on lysine-199 were identified by HPLC–MS/MS. Studies have shown that there are crosstalks between different kinds of modifications, which can be divided into two categories: positive crosstalk and negative crosstalk (37). Positive crosstalk means that one of the PTMs serves as a recognition signal for the addition or removal of the second modification, such as phosphorylation-dependent ubiquitination and phosphorylation-dependent SUMOylation. A negative interaction can be expressed as the competitive modification of the same modification site by two or more modifications, and the second modification may fail when the first modification occupies a specific PTM residue (38), such as the acetylation and ubiquitination of p53 (39). In this study, we validated the existence of acetylation and ubiquitylation on lysine-199 of N-Myc by synthetic peptides and put our effort into studying whether this PTM crosstalk can regulate the protein stability of N-Myc.

A variety of PTM regulation-related enzymes have been reported to participate in the regulation of the stability of the N-Myc protein by regulating its PTM. For example, as a ubiquitin ligase, HUWE1 can mediate the ubiquitination of N-Myc and target its degradation *via* the proteasome to decrease its stability. The overexpression of HUWE1 in NB cells can shorten the half-life of the N-Myc protein, resulting in inhibition of cell proliferation and promotion of neuron differentiation in *MYCN*-amplified NB cells. Thus, HUWE1 can be used as a drug target for the treatment of *MYCN*-amplified NB (11). In contrast, USP7, as a deubiquitinating enzyme that interacts with N-Myc, has the opposite impact on N-Myc compared with HUWE1. Its small-molecule inhibitors can promote the degradation of N-Myc *in vivo* and *in vitro*. The degradation of N-Myc inhibited the malignant biological behavior of *MYCN*-amplified NB cells, but the specific residues of these PTMs remain unclear (12). Our HPLC–MS/MS results of N-Myc co-IP identified several PTM regulatory enzymes that may interact with N-Myc, providing potential therapeutic targets for regulating N-Myc protein levels.

We then discovered that p300, a histone acetyltransferase that has been reported to mediate the acetylation of both histones and nonhistone proteins (14, 40), was a potential N-Myc-interacting protein and can regulate the protein level of N-Myc by regulating its acetylation and ubiquitination level simultaneously. Studies have also shown that p300 can regulate protein stability and activity by modifying the balance of acetylation and ubiquitylation of other target proteins, such as ALX1, HDAC1 (41), PRMT1 (42), XBP1s (43) and SIRT2 (44). In this study, we found that p300 interacted with N-Myc protein, and the amino acid 1 to 123 of N-Myc was crucial for the interaction. Furthermore, CPI-637, a p300 inhibitor, can modulate the stability of N-Myc by regulating its PTMs but had no effect on the function of N-Myc as a transcriptional activator. Thus, our results revealed that p300 could be used as a potential target for regulating the N-Myc protein in *MYCN-*amplified NB. Recent research revealed regulation of p300 on N-Myc mRNA *via* modulating H3K27ac and therefore N-Myc transcription (45), while our study focused on the PTM, protein stability, and degradation regulation of N-Myc mediated by p300, providing another mechanism of the regulatory effect of p300 on N-Myc expression. Also, the relation of p300 and c-Myc has been reported in 293T, osteosarcoma, and B-cell lymphoma cell lines, indicating that p300 can bind to c-Myc and regulate its stability (46, 47, 48), whereas p300 showed limited effect on c-Myc expression in NB cell lines based on our results.

The present study still has several limitations. For example, not all identified PTMs and residues were validated by synthetic peptides. We focused only on the PTMs on lysine 199 of N-Myc and its effect on N-Myc protein stability. The function of the remaining identified PTMs needs further research.

Taken together, our study discovered several potential PTM residues and interacting proteins of N-Myc, providing novel biological information and new directions for further research targeting N-Myc through PTM regulation. This study also revealed the interaction between p300 and N-Myc, and the regulation of p300 on the PTM level and protein stability of N-Myc, making p300 a potential druggable target for *MYCN-*amplified NB.

## Data Availability

The data generated in this study are available upon request from the corresponding author. The MS proteomics data have been deposited to the ProteomeXchange Consortium (http://proteomecentral.proteomexchange.org) *via* the iProX partner repository (49) with the dataset identifier PXD033457. The public data analyzed in this study were obtained from Gene Expression Omnibus at GSE45547 and GSE49710.

## Supplemental data

This article contains supplemental data.

## Conflict of interest

The authors declare no competing interests.

## Figures and Tables

**Supplementary Figure 3:**