# Supplementary figures and images for: P300 Interacted With N-Myc and Regulated Its Protein Stability via Altering Its Post-Translational Modifications in Neuroblastoma
Source: Mol Cell Proteomics. 2023 Jan 26;22(3):100504. doi: 10.1016/j.mcpro.2023.100504 (PMC9984901; doi:10.1016/j.mcpro.2023.100504)

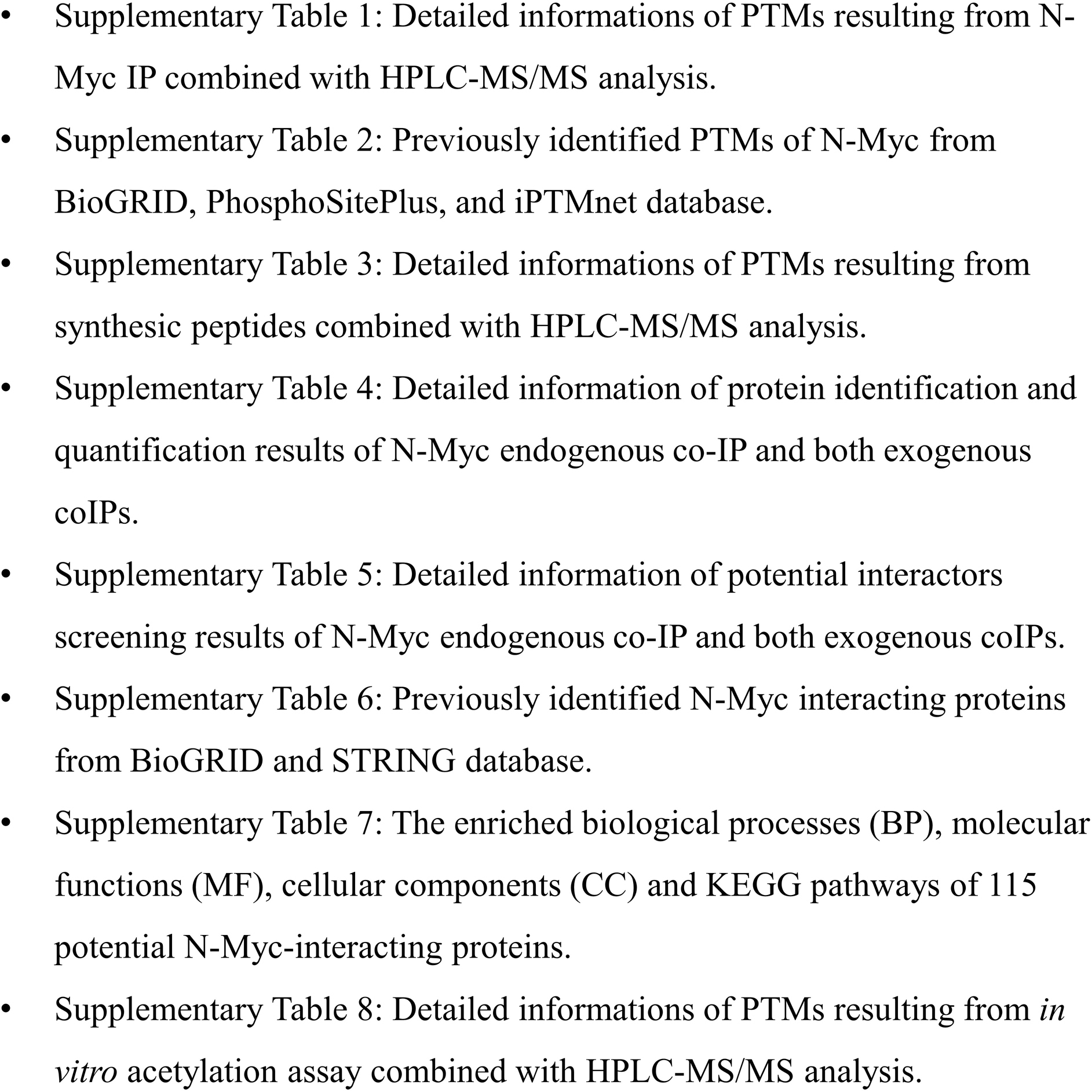

Supplement: Supplementary Table Legends [file figs4.jpg]
